# Book Reviews

**Published:** 1805-12

**Authors:** 


### CRITICAL ANALYSIS
OF THE
### RECENT PUBLICATIONS
ON THE
### DIFFERENT BRANCHES OF PHYSIC, SURGERY, AND MEDICAL PHILOSOPHY.

---

*A System of Arrangement and Discipline for the Medical Department of Armies.* By ROBERT JACKSON, M. D. 8vo. London 1805.

THE name of Robert Jackson, whether associated with Medicine or with armies, always arrests our attention, and we never find ourselves disappointed. A constitutional diligence matured by habit, a quickness improved by vast opportunities of practice, and a strength of judgment comprehending all those various combinations which are sufficient to confuse a weaker mind, have in a manner formed him for those intricate enquiries which are particularly important in the present state of his country. When to these we add an integrity which nothing can corrupt, and a boldness of expression which is only controuled by the laws of politeness, or by an innate sentiment of benevolence, we cannot but congratulate the soldier and the commonwealth on the task such a character has imposed on himself.

After a Dedication to the Officers of the Army, a Preface follows, containing an apology for thus appearing before the public on account of the little notice which had been taken of various applications to the Treasury. From a conscientious belief that large savings might be made of the public money, and the diseased or wounded soldier be better provided for, he proceeded to arrange his materials, so as to form a digest or System of Discipline for the Medical Department.

"The motive which prompted the design is pure; the purpose disinterested; the rule of execution unreserved. But, as the author was aware that motives are not always fairly interpreted, and, as the idea of offering something new implies the supposition of error existing in that which obtains, he thought it proper that an opportunity should be furnished to the confidential officers of the state, of examining statements, of ascertaining truths, and rectifying abuses in silence,—or without such exposure of facts as the formal publication of a book demands. Such rule of conduct his sense of duty commanded; and in prosecution of this impression, he intimated several months ago to the Chancellor of the Exchequer, (whom he conceived to be the proper organ of communication on this occasion, as being the first minister in His

 Majesty's

Majesty's councils, and the immediate steward of the national treasure,) that he (the author) had arranged a plan of medical management for the use of the army, which he was ready to submit to the examination of such persons as the Chancellor of the Exchequer might direct; supposing such to be persons competent to judge of the practicability and efficiency of the plan. If the value of the plan was tried and ascertained, the public might thus reap the benefit of the suggestions in the full extent, as rendered master of the power of applying them in practice after the manner that might seem most suitable. It was stated in this notice, communicated in a letter addressed to Mr. Pitt, and left at his house in Downing Street, that two-thirds of the means provided for the uses of the army employed on foreign service, especially during the course of the late war, was positively superfluous, as exceeding the just wants of the occasions,—the proofs incontrovertible. As Mr. Pitt did not deign to acknowledge the receipt of the letter, even by one of his under secretaries, the author, left without any official instruction respecting the most eligible mode of applying the fruits of his labours to the public benefit, felt himself under the necessity of putting his manuscript into the hands of a printer; for, with the conviction that two thirds, even with the conviction that one third of the means prepared for the use of the medical department of the British army, might actually be saved to the state, he would have deemed himself culpable to the nation had he withheld his communication."

The work itself is divided into five chapters. The first on the Constitution of a Medical Staff. The second on Military Hospitals. The third on Medical Management in Hospitals. The fourth on Economical Administration. The last contains a general Recapitulation of the Whole. At the end of each chapter are subjoined copious notes, illustrating the various reasonings and arguments, by facts universally admitted, well authenticated, or which passed under the author's own eye.

To render the first inquiry more perspicuous, and in some measure to introduce the main scope of the work, the first chapter contains a statement of the constitution, qualities, number, rank, and pay of a medical staff for a given military force. After a few words on the importance of the subject, Dr. Jackson, with his usual acuteness and accuracy, offers a short historical detail of the mode in which the health of soldiers was attended to in ancient and modern times. Of this we have only a slight sketch in the text, the more minute detail being referred to notes at the end of the chapter. Though this method is well calculated to preserve the uniformity of the work, we shall be obliged, in order to render our extracts intelligible, to incorporate some of the notes with the text. The following is the detail of the British medical arrangement in war.

"The British nation, which is often engaged in war, and which has often fought with a brilliant success in the field, does not claim claim an equal share of praise for the correctness of its military arrangements. The medical system adopted for the purposes of the army has fluctuated extremely; and it still fluctuates. In the earlier part of the last century, the medical provisions, constituted for the use of British troops acting in foreign countries, like the medical provisions of other warlike powers of the time, appear to have been very inadequate to the needs: the estimate of the soldier's value had not then been duly made. Whether the Duke of Marlborough was not sufficiently interested about the fate of his sick and wounded from conviction of the importance of the concern; or, whether he was not enabled, in defect of means, to afford the necessary assistance to those who suffered in misfortune, it has been often said, and it is believed to be true, that while the actual slaughter of Marlborough's battles was great, many, who might have been saved, perished in the field, in want of timely assistance from the surgeon. The voice of humanity was heard, or the interests of the state were better understood in the succeeding period. Earl Stair commanded on the Continent under His Majesty George II. and, during his command, such arrangements were effected with respect to the care of sick and wounded, as give more real value to his character than the fame of many victories. The value of health and the importance of the life of the soldier still rose in estimation. In the following continental wars, the hospital department was extended, with a view of better preserving the lives of men. The intention was good; the effect did not correspond with the intention. The hospital staff consisted of men of ability, and there is reason to believe that they did their best; yet the general hospital was esteemed to be the destroyer of the army; it was even noted by military officers of unprejudiced observation, who served under the Duke of Cumberland and Prince Ferdinand, that, where troops, trusting wholly to their regimental resources, were so circumstanced as not to have connexion with general hospitals, the loss by death was proportionally less than in the opposite case. Such was said to be the fact on the continent, under the rule of distinguished commanders and celebrated physicians; the practice was tried, and the effects were proved not to be different in America, in the American war. The British army was there well appointed in all respects; the hospital staff was numerous, and, upon the whole, well selected; the general hospitals were rarely crowded with sick beyond a just number, so that there was rarely any mortality from the operation of adventitious causes of contagion; yet, even where circumstances were so favourable, it may be said, without risk of incurring error, that, as the cure was more tedious, so the mortality was greater in proportion to numbers, in general hospitals than in the hospitals of regiments, though the latter were not always well equipped with necessaries, and sometimes could not boast of able medical officers; —this was verified wherever there existed means of making comparison.

 "The

"The British general hospitals, which were dormant after the peace of the year 1763, expanded considerably in the progress of the American war; they swelled rapidly, and burst forth into an enormous production at an early period of the late war. It may be thought necessary to notice in this place, for the sake of connexion and illustration of effect, that a Board was appointed in the latter end of the year 1793 for the management of the medical concerns of the British army. It is observed in ordinary life, that new men often solicit notice in their sphere by the adoption of new measures. The Board newly constituted, acting with the impulses of other men, attempted to distinguish itself by organizing a medical code on new foundations; this, it is natural to suppose, was to catch the impression of its masters. These, as practitioners in civil life, acquainted with nothing in the circle of medicine beyond the limits of the city of London, conceiving a general hospital to constitute the palladium of an army's health, formed the design of extending the sphere of military hospitals, more strictly speaking, of constituting general hospitals as the main instrument of medical effect for the purposes of the military force. The general hospital was thus considered as the great theatre, designed for the reception of military sick and wounded. The members of the medical board, as not being bred in the army, had no knowledge of regimental surgeons; regimental surgeons were consequently undervalued and overlooked, physicians and surgeons of the regular schools being held, in the opinion of the new chiefs, to be the only persons competent to act in military hospitals. The admission of the principle called for the adoption of a new measure, viz. the creation of new officers of the privileged classes. The regimental surgeon, not known to the members of the medical board, and scarcely permitted to make himself known by his knowledge and exertions, perhaps not enrolled at Surgeons' Hall, or not a pupil of a London hospital, not a member or licentiate of the College of Physicians, and not eligible to become so, as not admissible to examination while holding his Majesty's commission of surgeon, &c. was now barred the expectation of attaining the higher hospital rank; a privilege which had formerly been open to him, and a distinction which was held out to him as a reward of his services and his merits. The regimental surgeon, so circumstanced, could scarcely fail of feeling himself degraded; most people will be disposed to admit that he was injured; he may thus be supposed to have lost a portion of his zeal. If barred the expectation of promotion and the hope of advantages by the rule now enacted by the board, he was even in a manner stripped of confidence in his professional ability; for, as general hospitals were destined to be the great theatre of military sick, the slighter maladies, itch, sore legs, &c. were only supposed to be suffered to remain in the regimental infirmary. It might be deemed invidious to go deeply into the causes of this arrangement; it is important to notice its effects on the health of the British army."

In

In the rest of the note our author enters at large into the disadvantages of general, in comparison with regimental, hospitals. He reminds us, that in acute diseases, on the first measures and the early application of them, depends the future issue of the case. That, in more chronic cases, the sick soldier, removed from his early acquaintance or *commilitones*, pines under the uninteresting scene before him, or takes every opportunity of indulging that sensuality which is now the only source of amusement for him. If by degrees he becomes habituated to his new quarters and mode of life, from that moment he is lost to the service; his moral character is depraved, his exertions are stifled, and his only anxiety is to perpetuate that indolence which is the natural consequence of the apathy induced by his situation. But this is not all; the means themselves are destructive of the end. "The sick and lame, (says Dr. J.) flocked to these depots by hundreds; they returned not till after a long absence; and when they did return, it can scarce be said that they returned by fifties." The sickness in the British Army is known to have been great in the end of the year 1794, and the beginning of 1795; the mortality was excessive in proportion to the number of sick. The establishment of general hospitals, the means adopted by mistaken kindness for the relief of the army, may be considered as the main source of this dreadful mortality. Bold as this assertion may seem, our author proves it to our satisfaction in a variety of ways. He shows that the cavalry, who could with more facility remove their sick, so as to connect them with their respective regiments, were much more healthy. When the retreat became more rapid, it was found necessary to hire, or press waggons from the peasantry, in order to transport the sick and wounded infantry along with their respective regiments. During this period, and under these apparent difficulties, the number of recoveries was much greater, and the relapses comparatively few. Our readers will not want to be told, that so important a fact is not suffered to rest only on the evidence we have produced. The subject is continued through several pages, and in a manner which, we trust, will induce those, whose business it is, to enquire minutely into this important question. Thus much we have thought it necessary to premise, before we offer to our readers what may be called the inference which is contained in the text.

"The mode of applying medical means regimentally is assumed in this place to be the best: it has the obvious approbation of common sense, and the testimony of every military officer's experience that it is so. If the principle assumed be admitted to be the best, the next point of consideration relates to the rule of forming an estimate of the kind and quantity of aid necessary for the medical and surgical care of an army of a given force. This may be supposed to vary in a small degree, according to the manner in which the troops are arranged by regiments or corps, or according as they are destined for service in native or foreign climates.

climates. It is considered as preliminary in all cases, that each separate or independent corps, whatever be the force of which it consists, be provided with two medical officers, in order that it may be enabled to act with its own means in the event of indisposition happening to one or other of its medical members. One surgeon and one assistant surgeon will be allowed, by every reasonable person of experience, to be adequate to the ordinary medical care of a battalion of one thousand rank and file, stationed in Europe during the times of peace, whether constantly in garrison, or occasionally in camp. The number stated is supposed to be the common complement of medical officers for a battalion of the force specified, stationed as alledged; but, as great benefit will evidently accrue to the military discipline of armies, if battalions, consisting of one thousand rank and file, be formed into regiments of three battalions, or, which amounts nearly to the same thing, if three regiments, each consisting of one thousand rank and file, be formed in brigade, placed under the command of a general of eminent character constantly present at his post; so, on similar grounds of reasoning, if a chief medical officer be allotted to a regiment or brigade so constituted, a similar benefit might be expected to be obtained for medical discipline, with a more equal diffusion of the blessings of the medical art than now obtains in the army. The appointment of such officer, who is necessarily supposed to be an officer of experience and professional skill, capable of connecting and binding all the parts of duty together, would be sufficient to render the proposed medical establishment of the army adequate to its needs in all common circumstances of service. It is presumed to be adequate in number; for, estimating the proportion of sick at one in ten, which is a high proportion among well-organized troops in European climates, there are provided seven medical officers for a regiment or brigade of three thousand rank and file. The care of three hundred sick soldiers divided among seven persons, at the allotment of forty-three patients to each person, cannot be supposed to be an oppressive duty to active and capable men; and, of such only, the army surgeons must be supposed to consist. Such a portion of duty, it is presumed, would not be thought to be hard by any one: it may even be added, that did the number of the sick, on certain occasions, actually amount to one in five, the requisite attendance, in its fullest extent, upon eighty-six sick soldiers in a well-regulated military hospital, where the diseases have probably a great similarity of feature, and where several of them are probably only of the slightest kind, or such as do not require a daily new prescription, cannot with propriety be reckoned a task of hard labour to an active man, particularly as it is a task which is not likely to be of long continuance. If the arrangements be methodical and correct, the duty will be light, as the numbers now stand: if the arrangement be faulty and and deficient, no increase of number will give just effect to execution.

"The estimate, which is given in this place, may be considered as the estimate of a just medical establishment for a regiment of three thousand rank and file. If the number stated, viz. seven medical officers, be sufficient for the medical purposes of a regiment of three thousand men, two hundred and thirty-one, the calculation being made upon the same principle, will be equal to the purposes of thirty-three regiments or brigades, consisting of one hundred thousand, or rather of ninety-nine thousand soldiers. If the sick be calculated upon a scale as amounting to one in ten, an army of one hundred thousand men produces ten thousand sick. The allowed medical staff, consisting of two hundred and thirty-one surgeons and assistant surgeons, is confidently maintained to be equal to the medical care of the number of troops stated, where hospitals are well arranged, stations permanent, and quarters fixed in a peaceable country. There are for instance only forty-three patients for each surgeon, which is an allotment of duty, sufficient to occupy the time and to engage the attention ardently, but not of the extent to be considered as an oppressive toil. In active war in the field, where there occasionally occurs a necessity of detachment, or in foreign stations and new climates, where there is reason to expect unusual sickness, it is deemed a wise and provident measure to add three extra assistants to each regiment or brigade. The most experienced of the battalion assistants may reasonably be supposed to be the fit persons to be selected for this duty: the office implies a responsibility; and it is held to be a step leading to a permanent promotion: it forms such addition to the common complement of regimental staff as is supposed to be sufficient to enable an army to meet the ordinary occurrences of war, or the pressure of sickness in unhealthy climates—without difficulty or embarrassment;—it is regimental means attached to a moving or moveable body.

"Armies frequently change their positions in the scenes of actual war; and, as there are sometimes found persons in armies, so disabled by wounds or other circumstances of sickness, as not to bear the fatigue of transport without inconvenience, pain, or danger, it becomes necessary, as being humane and even economical of life, to establish hospitals in places of security, for the more safe and ready accommodation of particular cases of chronic disease or dangerous wounds. Such being the rule of arrangement, one surgeon and three assistants, one physician and three assistants, may be thought to constitute a sufficient provision of medical officers for such kinds of sickness as cannot so properly be confined within the regimental circle,—in a division of an army consisting of fifteen thousand rank and file. According to this rule of calculation, an army of one hundred thousand men requires six physicians and eighteen hospital hospital assistants or mates, six surgeons and eighteen assistants, extra of the regimental or brigade addition, estimated above as the just allowance for the purposes of war or foreign service."

This important subject is further considered in every point of view, and after stating the number and rank in the profession of medical officers sufficient to take the charge of a regiment, of a brigade, or of an army, in time of actual service, the chapter concludes with an enquiry into the best mode of education for army physicians—the rank they should hold regimentally or in brigades—generally, or in their respective regiments; their pay; and, lastly, the discipline and forms of medical duty, which they should submit to and inculcate.

The second chapter on *Hospitals*, contains a most exact description of the best mode of constructing, equipping, and governing such institutions. This subject has been so frequently discussed of late, that we could expect but little novelty from our author. However, the last department, as directed to the military, contains many useful instructions. But the most interesting part is contained in the notes, in which, a short historical detail is given of the different military hospitals which have sprung up since, or during the late war, and some of which may be said to be already abandoned.

The third chapter, on medical arrangement, is entirely new, excepting such suggestions as have appeared in our author's former publication. Every thing is comprehended which can relate to the preservation of life, the comfort of the sick, the means of providing for the most minute, though oftentimes not the least important offices, as well as the extension of medical knowledge, by enforcing an habitual accuracy of discernment, and the advantage of generalizing facts. Nor are these suggestions confined to hospitals, or even to the calm period after engagements—the station for the surgeon and all others, who can be instrumental in the humane services of the wounded, is assigned for the time of action—the principle and discipline of nurses and orderlies, the provision of medicine and their expenditure, the estimate of the attendant expences; in short, whatever can be comprehended under medicine or surgery, in any situation of an army, is minutely and accurately provided for.

Chapter the fourth is confined to the economical administration. Here our author takes an accurate review of such servants as are necessary, and such as have been introduced for want of a just knowledge of the movements and discipline in armies. Among the latter, he reckons the matron, whom he considers as necessarily too good, or too bad for her office. If she is a woman well educated and suited for the superiority she is to assume over the nurses, her office soon becomes irksome, and the society she is confined to, renders her only anxious to get over the necessary detail, and retire from a scene, to which she ought to be always attentive. If she has accepted her office with a view view to emolument, such will be her only object; and instead of superintending the economy of the house, she will suffer others to purloin, that she may not be detected herself. All the necessary provisions of the house, even to the captiousness of sickness, Dr. Jackson not only admits, but dwells with a most laudable anxiety on the means of inducing the invalid or convalescent to derive every benefit that arises from delicacy and variety of viands. A method is proposed of simplifying accounts, which is illustrated by a table, enabling the most ordinary capacity to fulfil with facility all the complicated offices of clerk and purveyor. All the checks and controuls hitherto imposed on hospital accounts are shown to be futile; and it will be scarcely believed, that the plan proposed is no less than that the soldier should be provided out of the stopping from his own pay with all these articles, yet receive, on returning health, a considerable surplus. It will be of course presumed, that particular cases may occur, requiring extraordinary aids.

Though we have read this part of the work with peculiar satisfaction, we must acknowledge that much of this has arisen from the contemplation of our author in his office as a good Samaritan. Dr. Jackson is not, perhaps, aware of all his own good qualities, and may expect to meet with the same dispositions, and the same capacity of action, in other army physicians and surgeons as he feels in himself. We would not, for a moment, suspect any want of tenderness in a medical man, however situated: but to accomplish what our author has done, requires not only benevolence and skill, but strength of body and mind; a courage which can only arise from, and be supported by, a conscious superiority, and a firmness which must sometimes submit to indignities, the more painful as they are undeserved.

The last chapter contains a general recapitulation of the whole. The language of this part is particularly pointed, and the subject as much compressed as possible; as a specimen of which, we extract the following concluding paragraphs.

"The economical administration appears, as well as the other concerns of British military hospitals, to be capable of a more correct arrangement than that which now obtains, both as perfecting effect and as saving the expenditure of means. It is capable of demonstrative proof, that the numbers of the servants of the economical administration may be diminished: the purveyors and matrons, as now constituted, may be entirely annulled as superfluous, if not embarrassing. The diets of hospital subjects, regulated by a correct scale, shew plainly, that the value of what is sufficient for the support of a man in health, is equal to supply the requisite quantity of what is suitable to a man in sickness, in ordinary cases of disease. If this be so, and the proofs are incontrovertible, all that which exceeds the just measure is waste. This, in British military general hospitals, appears, on some occasions, to have been prodigious: a reference to the numbers numbers of sick, compared with the expenditure of means, shews the precise quantity. The arrangement, exhibition, and control of accounts is another of the objects which is considered to be of much importance; but it does not yet seem to be so digested as to touch the useful point. The control alluded to is a control of figures,—the transcript of distant transactions, not of things verified by direct inspection on the spot. This is plain; the evidences incontrovertible. The medical inspection of hospitals has also undergone a great alteration of late years, or rather it has arisen as an entire new creation. If the subject be well considered, it will probably be discovered that it is not constructed upon a principle to produce an uniform and systematic effect;—the instruments are numerous, and, as they have not been formed on one model, their views may reasonably be supposed to be various or discordant. So constituted, they cannot be expected to produce uniformity in action; and it is difficult to say in what degree they are to be regarded as useful. It is undeniable that the cheapest and most effectual way of executing the medical business of armies, is by the selection of able regimental surgeons, —men who know their duties in all their extent. The process then moves on correctly without the inspector: it is a vain expectation to hope, if the surgeon be radically defective, that he will be instructed or enlightened by the unimportant or cursory visit of the inspector's deputy.

"If the subject of hospital management be viewed in all its details, it will be readily admitted that it is capable of improvement; and, if the importance of the subject be duly considered, the improvement proposed will strongly command the attention of statesmen. If there appear evidence, and it is believed the evidence is demonstrative, and may be verified in a reference to the authentic documents which are lodged in public offices, that two thirds of the means provided for the use of the sick in mostof the expeditions which were undertaken since the year 1793 were superfluous—as exceeding the just needs;—viz. the medical officers so numerous, as not to have an opportunity of acting fully and effectively in their stations; the stores of medicine, &c. so excessive in quantity as to decay and perish in the magazines before there occurred an opportunity of applying them to their purposes, there exist strong reasons for a reform; and the plan now recommended, though not the most perfect that might be devised, will stand excused in its motive,—and perhaps escape with inferior censure for the conduct of its detail. It will not be deemed impertinent by those who regard the interests of the public with a just eye: the subject is a national concern, and the investigation is open to every honest subject of the nation. If error exists, its operation is injurious; if a remedy be attainable, it is a duty to make it known. The reform of error is not an innovation in the real meaning of things, though it has been styled so in the language of those who are prejudiced; nor is the man inimical inimical to his country and the interests of humanity, who suggests such arrangements, as are calculated by the correspondence of things with each other in their natural and just relations, to move with harmony and produce a correct effect. Such arrangement, while economical of means, produces a just and permanent action in all its stages. It is such as the author has attempted to attain.—The idea of the system, which is now explained and made public, arose at an early period of life; it was traced through many varying scenes of service, pursued in spite of difficulties and opposition, till such evidence was obtained of its truth as may be considered to be demonstrative in all its steps. It is finished, and it may be necessary to add, that as the subject was prosecuted as an object of study, the results are now given to the public as a command of duty. The author has no private view nor prospect of advantage from the fruits of the publication: he gives it freely without expectation of reward; and, acting honestly and independently, though placed in an humble sphere of life, he is neither solicitous of praise nor fearful of blame in the course which he pursues."

Such is the nature of this most important work, the value of which it is impossible to estimate. In distant expeditions, their whole success often depends on the health of the soldiers. That this may be preserved, at least under many unfavourable circumstances, we have many proofs; but we do not recollect a writer, who has so faithfully explained the causes of that dreadful mortality, which so often prevails in climates, to which European constitutions are habituated, or that has shown such persevering industry in finding a remedy, the success of which, he has proved in his own practice. We hope all the valuable instructions contained in these sheets will be duly appreciated, and that our Author will have the happiness which attends virtuous and intelligent minds, when they see their labours justly considered, and mankind benefited thereby.

---

*Observations on the Simple Dysentery and its Combinations, containing a Review of the most celebrated Authors who have written on this Subject; and also an Investigation into the Source of Contagion in that and some other Diseases. By* William Harty, M. B. 1805.

In some preliminary observations, the author informs us, that from all he has observed and read relative to dysentery, it appears to him, as to Dr. Cullen, a single disease. But he differs from the Professor in considering dysentery in its simple form, without pyrexia or contagion, as the true disease; whereas Cullen has made pyrexia a part of its character. Dr. Harty's object is to show, that whenever fever, whether of typhus, or intermittent or remittent kind, attends dysentery, it is an accidental circumstance; that, consequently, the contagious nature of the disease depends on its its combination, and that no form of dysentery is contagious, excepting that which is combined with typhus.

In the progress of the work, the author informs us of his intention to show that this contagious character of dysentery is equally applicable to other diseases under similar circumstances. His object in this he professes to be principally to direct the attention of others. In this we heartily wish him success. Enquiries more minute, descriptions more accurate, and distinctions more precise, have always been wanted in medicine; and, until they are accomplished, it will be vain to look for solid improvements in so complicated an art. How far our author has improved the accuracy of medical reasoning in this disease, will appear as we proceed.

The first chapter being superscribed *simple dysentery*, we should have expected a regular history of the disease, had it not been that we are before told, this is no part of the author's intention. The chapter consists of a diligent digest of the opinions of the most celebrated authors; from all which a conclusion is drawn, that fever is not a necessary part of the dysenteric character. All the remarks contained in this chapter are judicious and pointed, but every reader will regret that the author is so costive in forming conclusions, which might connect the various threads of which his reasoning is composed. It may be urged, that all this is referred to the conclusion of the work. This may be enough for all the purposes of the logician; but the common reader expects to meet with well established inferences, ready drawn for him, from all the parts as they occur in order; by help of these, he is relieved from the retention of a vast burthen of facts, feels a conscious satisfaction in so far understanding the object of his author, and looks forward with patience to the accomplishment of the whole. However, from the following extract, which contains a very just reproof against the attempt at reducing to nomenclature a class of phænomena in which so few men agree, our readers will form some judgment of our author's views.

" When we have it asserted that dysentery is a " Pyrexia contagiosa," I presume it is meant that the disease is attended by* fever, not merely symptomatic, but peculiar to that affection, and that its contagion is also of as distinct and independent a character. Now, that it is not necessarily attended by idiopathic fever, the very nature of that fever, when present, and its total absence on other occasions, plainly shew; and that it is not of itself contagious will hereafter appear; as also, that when, in consequence of a certain combination it becomes so, that contagion is not peculiar to this disease.—I trust then I may be allowed to conclude with

" * When we hear authors, at one time, calling this fever inflammatory, and at another time, intermittent, remittent, or putrid, surely we must suspect that the disease can scarcely be possessed of an idiopathic fever, peculiar to itself, inasmuch as such fevers seldom admit of this variety."

with an assertion, which as we advance, will be receiving additional proofs, namely, that the genuine dysentery is unattended by any but symptomatic fever, proportioned to the violence and severity of the disease itself; while, at the same time, as shall hereafter appear, it is capable of combining, under appropriate circumstances, with intermittent, remittent, and typhus fevers.

"If then it be admitted, that the symptoms already enumerated can, independent of "Pyrexia contagiosa," constitute a distinct and well-defined disease; and for admitting this, we have sufficient grounds in the experience of every year, and in the testimonies of creditable writers, shall we refuse it the name of dysentery, to which it appears entitled, merely because it does not chance to agree with a scholastic disposition? Surely we should not: yet that some have been, and still are governed by it, in judging of the disease, an incident which occurred, while I was a student at an academy of deservedly high repute, will too plainly shew: A patient came into the clinical wards of the Infirmary, with every mark of simple dysentery. I examined her: she had no symptom of fever, and it was not possible to trace the disease to contagion. The clinical Professor, a gentleman of considerable merit, and for whose attentions I ought to feel grateful, visited the wards soon after, examined this patient very carefully, and in his questions was most particular relative to the "Pyrexia contagiosa;" finding, however, that the former did not exist, and that there was no ground for believing in the agency of contagion, though the other marks of dysentery were all present, it would appear by the result, that he felt himself in some dilemma, how to decide between the disease and the definition; though he had no hesitation whatsoever, as to the practice he should adopt on the occasion. At a subsequent lecture, he pronounced the disease to be a diarrhœa, while he said he would treat it as a dysentery. In consequence of this decision, purgatives, &c. were administered, and after a time the patient got well.—Strange, that our reason should be so warped by a definition, as to induce us to call a complaint by one name, and treat it under the name of another."

The next section of this chapter is on the analogy between dysentery and rheumatism. In this it is urged, with much ingenuity, that the latter disease, though usually considered as inflammation of the muscles, is most probably inflammation of the mucus membranes, and therefore that the mucus membrane of the intestines may be affected in a similar manner from similar causes. We are not perfectly satisfied with our author in many parts of his reasoning on this subject, though we are more inclined to consider not only these two diseases, but many others, as analogous. Inflammation, whether chronic or acute, wherever situated, has its effects modified by the original actions of the parts; and a change of the seat of these actions is not uncommon, not only between dysentery and rheumatism, but many other complaints; nor can we perceive that exact analogy which our author wishes to establish between between increased secretions in these two diseases, beyond what happens in inflammations of any other secreting surfaces.

In the second chapter, on the combinations of simple dysentery with intermittent and remittent fever, Dr. Harty first enters into a distinction of the manner in which two diseases may exist at the same time, in the same constitution. "There are, (says he) two species of combination of disease; the one is such a combination of the character and features of each, whereby they seem to incorporate together, to possess one nature, and exercise one dominion; the other is an accidental union of two or more diseases in the same individual, which are naturally, and continue to be mutually independent of each other; the latter are in general local diseases, as itch, syphilis, herpes, &c. or one, or more of them may be local, while a third is a general affection of the system. But then the local complaints arise from specific contagion, as in the combination of typhus with itch, &c." page 39.

All this is a little confused; but our author's meaning is, that two local complaints may exist in different parts of the body at the same time, and also that a local complaint may exist with a constitutional complaint; but that two constitutional complaints cannot exist at the same time, without so altering the form of each that they shall become one disease. Hence, in seasons or situations where intermittent or remittent diseases prevail, dysentery may likewise be epidemic, and the two diseases be so far combined as that one may appear a symptom only of the other.—This, it is shewn, is the origin of the *febris introversa* of Sydenham, and also the remittent taken notice of by Clarke, in which a rapid dysentery is one of the most dangerous symptoms. Morton also refers more obscurely to the same subject. But our author has no difficulty in showing that Cleghorn, and most of the modern writers on diseases of camps and of warm climates, were sensible of the fact, insomuch that some of them treated dysentery with bark in the manner of a common intermittent. It should, however, be remarked, that Dr. Cleghorn considered the intermittents of Minorca as contagious; and in this opinion he seems supported by Dr. Rush. We mention this not with a view of giving any opinion on the subject, but to show how unsatisfactory the distinction between a contagious and merely epidemic disease remains even among the most accurate observers of those diseases, to which, at present, the attention of the whole world is directed.

The next chapter is dedicated to the subject of combination of simple dysentery with typhus or malignant contagious fever. This the author very properly introduces, in a manner to arrest the attention of the reader, and to force on his recollection all the various distinctions to which he has previously alluded.

This, it is remarked, "is the combination which is never free from symptoms of fever, and to which alone the property of contagion belongs: this is the true "Pyrexia contagiosa:" it is after this this form that Cullen has framed his definition of the disease; it is this form which has always inspired such horror at the name of dysentery. The strong features of such a combination are very decidedly marked in the histories of the most celebrated epidemics of this disease; and between this and all other forms, the reader may trace the most essential differences in their access, progress, and termination; in the danger attending them, and in the mode of treatment, which success warrants in each."

After this follow two sections; the first, containing proofs from the symptoms that such is the true contagious dysentery; the second, proofs from the general history of the disease. In both these our author shows much industry in research and acuteness in judgment.

The fourth chapter contains a general inference from these premises; and as we conceive most of our medical readers will be sufficiently satisfied by their own recollection, how much the different writers on dysentery have differed on the subject of its contagious property, we shall not be thought abrupt in transcribing our author's conclusion.

"Any person in the least conversant with the writers on Dysentery, must have perceived the most striking contradictions in their belief and assertions on the subject of its contagion: he will find some denying it in toto, others as positively declaring it to form one of the strongest features of the disease, while a few, more moderate in their sentiments, though more undecided in their opinions, may be observed to waver between these extremes, and who, in admitting, or excluding, contagion from some particular epidemic, do not venture to pronounce it absent, or present, in all others, nor attempt to point out the particular cases, in which we might expect to see the influence of that agent exerted. If we look to authority as the basis on which we are to rest our opinions (and to what besides this, can we resort in the decision of such a question?) in the circumstances just described shall we find our criterion situated; and what is worse, should we be disposed to weigh the opposite authorities against each other, the names of respectability appear so evenly balanced on each side the beam., that it will not, it cannot preponderate either way. Thus, if we suppose that the authors who have written on Dysentry, described under that name the same unvarying and identical disease, do we perceive that on the ground of authority we can arrive at no decision, inasmuch as the opposite assertions destroy each other. But if it be true, as I have stated, that there are forms of this disease very different in their nature and characters, and that these authors, as has been shewn, under the same name of Dysentry, described these its different forms, then may some hopes be entertained, that we shall be able to reconcile the most direct contradictions and opposite assertions, without injury to the character of either party; for as each described the disease he met, with the accuracy and fidelity of an historian, they must necessarily have differed from each each other in their description of the disease, and in their opinions respecting it, because while one found the disease in its simple state, others saw it only in its state of combination. That such must have been the case, ought to have been supposed, from the very circumstance of men, well known for their veracity and talent for observation, *appearing* to contradict each other in so direct a manner, and to form the most opposite opinions on the *same subject;* and that such was the case, the facts already adduced have, I trust, been sufficient to satisfy any unprejudiced mind. If such then be the case, how grateful must it be to the mind of every man to think, that he may on this, as on other occasions, give some credit to the assertions of his fellow-men, without incurring much danger of running counter to the truth, or of falling into any material error.

"The extremes of opinion entertained on the subject before us, are, on the one hand, that the disease is never contagious, and on the other, that it is always so, and that this property is owing to a specific virus. On this, as on all such occasions, we shall find the truth of the adage, medio tutissimus," for between the extremes of opinion now mentioned, a proposition may be stated which shall embrace a part of each, which shall be agreeable to fact, and which shall meet with a general concurrence. The proposition, which it is my intention to uphold, has been already often alluded to, and it is this: That THE SIMPLE DYSENTERY IS OF ITSELF NEVER CONTAGIOUS, NOR THE INTERMITTENT AND REMITTENT FORMS OF THE DISEASE; That THE COMBINATION WITH TYPHUS IS ALONE POSSESSED OF THAT PROPERTY, and that THAT PROPERTY ORIGINATES, NOT IN ANY VIRUS SPECIFIC TO DYSENTERY, BUT IN THE CONTAGION OF FEVER.

"The truth of these propositions I shall endeavour to establish in the following manner: We have already seen who the authors are that describe the disease in its different forms: of these and of a few others, not as yet referred to, I shall take a general survey, and after stating the sentiments of each on the subject of contagion, and contrasting them with each other, we shall, I believe, find reason to conclude, that such authors as describe the disease either in ITS SIMPLE FORM, OR IN COMBINATION WITH INTERMITTENT AND REMITTENT FEVERS, uniformly pronounce it, NOT CONTAGIOUS; while those who met it in COMBINATION WITH TYPHUS FEVER, as regularly and decisively declare it TO BE SO. The inference unavoidably consequent on such a conclusion, must be, that the truth of the propositions, above stated, rests on the fairest and strongest grounds.

"To proceed therefore, I shall begin by considering the sentiments of that man, whose definition of this disease has had no small share of weight in influencing and deciding the opinions of others; and should I be able to shew cause, why he was naturally led to embrace such sentiments, without necessarily inferring that he was correct in so doing, I hope that his authority will no longer stand in the way of truth, nor exert improper influence over the minds of others.

others. This is my only motive for taking any notice here of the opinion of Dr. Cullen relative to the Contagion of Dysentery, inasmuch as it has been, and is still my intention (with the exception of this deviation) to confine the present survey to original observers of the disease, to men who described it as they saw it, and not as they found it described; among these Dr. Cullen does not, nor pretends not, to rank, for he spoke of the disease not from his own, but from the experience of others. On this account his authority is on a level with those, whose writings on the subject he has consulted, and whose opinions respecting it he has adopted; and as it was his uniform practice, after giving the definition of any disease, to enumerate those authors he had consulted, so he has himself furnished us with the means of judging of the weight that should be attached to his opinions relative to this disease. What these opinions are has been already, in great measure, pointed out: they are pretty plainly specified in the first sentence of his definition, which states the Dysentery to be a "Pyrexia contagiosa:" he even goes so far as to say, that he thinks it doubtful if the application of cold does ever produce the disease, unless the *specific contagion* has been previously received into the body. v. vol. III. p. 119. Now the reason why Dr. Cullen has adopted these opinions, will be pretty obvious, after enumerating the names of those authors to whom he has referred on the subject of Dysentery: I need but mention Pringle, Degner, Roederer, Zimmerman, Grimm, Helwitch, Bontus, Cleghorn, &c. &c.; most of these, have already been quoted, and all of these excepting Cleghorn, who described the intermitting variety of Dysentery, will be found on an examination of their writings, to have seen the disease in its combination with Typhus, which they all with one voice (making the same exception) pronounce to be contagious. Had these been the only authors, whose opinions or observations relative to this disease could be relied on, Dr. Cullen would have been perfectly justified in the definition he has given of it, but such is far from being the case; and had Cleghorn stood single, it ought to have been shewn, why he has said nothing of the contagious property of the intermitting species of the disease. As it stands, we may perceive that Dr. Cullen has given us a definition of the combination with Typhus, in place of the definition of the disease itself."

This chapter contains two other sections, in which the subject is further enforced.

The fifth chapter is on the treatment of Dysentery. From the practice recommended by different authors, the proofs of these various states of Dysentery are further enforced; the various remedies are stated seriatim, and their application in the different forms and combinations of the disease judiciously marked. Throughout the whole we conceive our author much more fearful of the lancet than is necessary; that its decisive good effects in some instances have at times too indiscriminately recommended its use we pretend not to doubt, but are not less convinced that the occasional

 occurrence occurrence of highly putrid symptoms at a later period of the disease, have rendered many practitioners constantly apprehensive of debility as the most formidable symptom to contend with. On this subject we have already given our opinion more at large, when we reviewed Dr. Wilson's last volume.*

The last chapter is on diseases analogous to Dysentery in the source of their contagion. These are, 1. Catarrh, 2. Angina Maligna, 3. Ophthalmia, 4. Erysipelas, 5. Malignant Ulcer, or Hospital Gangrene, 6. Puerperal Fever. After which the author concludes with some general remarks, which, if it were necessary, would further convince us of his industry, modesty, and genius.

Having thus, as far as our limits will permit, gone through this work, we shall find no difficulty in recommending it to our readers. It contains, unquestionably, a larger mass of evidence than is any where else to be found of the various species of a disease, which though but little known in the common walks of civil life, is among the most formidable in camps, in armies, and sometimes in the abodes of poverty, and its attendant wretchedness. We sincerely hope our author, before another edition is called for, will be able to add his practical remarks to what he has selected from others. He will then find, experimentally, how difficult it is to make these remarks in a satisfactory manner, and how frequently he is obliged to doubt the faithfulness of such authors as are the most disembarassed in their histories and inductions.

As to the theory contained in the work, it is evident that Dr. Harty does not consider it new. The number of authorities quoted in every page, must have convinced him that if the writers he refers to, did not make exactly the same distinctions as he has done, many of them were aware of them, and conceived them well understood. This, however, does not lessen the value of our author's investigation. Some of the best received writers who, are most commonly referred to, certainly overlooked the distinction. The whole herd of nosologists, with Cullen in their van, and bringing forward all their forces, are very much confused for want of a practical knowledge of those forms of the disease which probably never occurred in their own practice. The last mentioned author was totally unacquainted with them, as Dr. Harty has shewn in several passages. Dr. Jackson, however, whom we should gladly have seen quoted more frequently, is very careful in marking all those various effects produced from similar causes. There is, indeed, a difference in the opinions of these gentlemen. Dr. Harty seems to consider typhus as necessary to render these topical diseases contagious: Dr. Jackson considers the tainted atmosphere of crouded hospitals, which at one time produces typhus, may, under other circumstances, produce those local effects which may afterwards end in fever, or fever may terminate in them. From the practical

* See Vol. xiii. p. 654.

practical knowledge which Dr. Jackson possesses, independent of all other considerations, we cannot but give him our decided preference; nor can we refrain from expressing our wish once more, that our author may be able to meet Dr. Jackson, as an antagonist, on equal grounds, by acquiring a practical knowledge of a disease, the history of which he has so accurately detailed from others. In saying this, we think it right once more to recommend the work before us, as the most valuable digest of all that has been written on this formidable disease.

---

*Cow-Pox Inoculation no Security against Small-Pox Infection.* By W. Rowley, M. D. *Member of the University of Oxford, the Royal College of Physicians in London, Physician to the St. Mary-le-bone Infirmary, Author of Schola Medicinæ Universalis Nova, the rational and improved Practice of Physic; and Public Lecturer on the Theory and Practice of Medicine, excluding false Systems, &c. &c. To which are added, The Modes of Treating the Beastly New Diseases produced from Cow-Pox, explained by Two Coloured Copper-Plate Engravings: as, Cow-Pox Mange, Cow-Pox Evil or Abscess, Cow-Pox Ulcers, Cow-Pox Mortification, &c. With the Author's certain, experienced, and successful Mode of Inoculating for the Small-Pox, which now becomes necessary from Cow-Pox Failure, &c.* Octavo, London, 1805.

This Title Page shows so exactly the intention of the author, that a commentary upon it may serve as a review of the work. First, it is a means of introducing our author's titles: *Member of the University of Oxford!*—It is usual for graduates, at any University, to specify their precise degree. Dr. Rowley, on account of "his long life, incessantly dedicated to the study and practice of physic,*" must, certainly, stand as Doctor of physic in that University.

---

* These words are taken from the Introduction, and the following Note is tacked to them, "As *Schola et Historia Medicinæ Universalis Nova*, the rational practice of physic, and other works testify; but with what success, must be submitted to the candid consideration of the learned faculty all through Europe. In the public medical lectures he has had the honour to deliver to numerous students and others of the faculy, it is well known that the *Boerhaavian*, *Cullenian* hypothetic systems, as likewise narcotic *Brunonian* visions, have been proved partly erroneous, and therefore inadmissible in the true practice of medicine. It has been attempted to raise the whole art on a more solid basis, by only admitting self-evident and undeniable facts from the science of anatomy, physiology, pathology, and *successful long experience* in practical medicine."

When a man writes in this manner of himself and his writings, it is impossible not to suspect him; but when he appeals to the learned faculty of all Europe, relative to the success of works scarcely known, but by the frequency with which they are advertised, we then understand that cow-pox, or any other popular subject, are convenient things to bring forward a name, which might be lost but for such opportunities. Thus we find Dr.

sity. But, his modesty has induced him to take a title, which any term-trotter may assume, almost as soon as matriculated. As to the works, the authorship of which he claims, we trust, no one will dispute his title to them. His *public* lectures too, have been too often advertised, for any one to doubt their publicity or the liberality of the author to his audience. If they will but have the goodness to hear him, it is well known, how indifferent he is to all pecuniary considerations.

We come now to the latter part of the title, viz. The mode of treating the beastly new diseases produced from Cow-pox, explained by two coloured engravings, as Cow-pox Mange, Cow-pox Evil or Abscess, Cow-pox Ulcers, Cow-pox Mortifications, &c. The history of the first of these engravings is a little curious. We have before remarked Dr. Moseley's apparent fondness of fun. "Dr. Moseley, who (says our author in a note) sensibly first exposed the errors of vaccination, saw the case of the ox-faced boy by my desire. He observed to me, that *the boy's face seemed in a state of transforming and assuming the visage of a cow.*" This was an arch thought of Dr. M.; but if he had a mind to make his brother antivaccinist ridiculous, he should have recollected his friend and neighbour, and not have suffered him to expose himself by publishing his own drolleries, and *illustrating them with a coloured engraving.* We happen to know something of this case, which proved nothing more than a common abscess, the breaking of which, like the breaking of Circe's spells, restored to the boy his natural countenance; and under the surgeon of the Bloomsbury Dispensary, is now healing like any other abscess. The history of the case will convince any one, that vaccination could have no share in the disease, trifling as it proved.

The facts we are told on which the propositions (the insecurity and subsequent danger of vaccination) depend, are extracted from Dr. Moseley, Mr. Goldson, Dr. Squirrel,* Mr. Rogers's and Mr. Birch's observation. All these we have already considered. Our author tells us, he has added above a hundred more. It was merciful not to add a thousand whilst he was about it; but when he is satisfied with such authorities, most of which have been already enquired into, it will not surely be expected of us to examine the remainder; nor, if we did, could we expect our readers to attend to the result. It would be strange indeed, if out of more

Dr. Squirrell and Mr. Velno, have their specifics against the effects of cow-pox; and in a short time we shall, perhaps, be told, that Mr. Reymer's cordial is a valuable remedy for these, as well as all other complaints.

* We are told, in a note, that Dr. Squirrel was formerly many years apothecary to the Small-pox Inoculation Hospital at Pancras; Dr. Squirrel never was at all connected with that hospital, he was for a few months apothecary to the Small-pox (but not the Inoculating) Hospital at Cold Bath Fields.

more than a million of people who have been vaccinated, the ingenuity of some, the humour and the malice of others, and most of all, the desire of consequence, which, more or less, attaches itself to all, should not be able to produce as many cases as Dr. Rowley has here brought forward. It would be still stranger if some real failures and well authenticated inconveniences had not occurred in a practice so widely extended; for what have we of certainty either in disease or health? Vaccination is not proposed but as the means of avoiding a greater evil, and who will pretend to compare the few untoward cases which have occurred with the numbers that have been always admitted by the warmest advocates for inoculation? But we have not yet got through our remarks on Dr. Rowley's title page—There yet remains to be considered—"The AUTHOR's certain, experienced, and successful mode of inoculating for the small-pox."—Aye, there is the business. If you wish for a certain, and (which we suppose means something more than certain) successful mode of inoculating, you must apply to Dr. Rowley, Member, &c. &c. &c.

All this may be collected from the title page, which some may think is as much as the author wishes the public to read, or rather is what he has taken care shall be read in every newspaper, and in all the diuretic corners of the streets. But we, who in our office of Reviewers, thought it our duty to peruse the whole, can assure our readers, that the Doctor will be greatly disappointed if they stop at the title page. The notes are for the most part not less important. They evince such intense reading, and so capacious a memory, not only in medicine, but in all the arts and sciences, as must astonish every common capacity. They show also what a great author Dr. Rowley is, and also what a great and experienced practitioner. One instance we have given of this already. The following shows how fortunate it is when the sick consult such a man as the "Lecturer on the art of physic excluding false systems."

"In my lectures," (says Dr. R.) "on the art of physic, both theoretical and practical, I have fully proved, that there is no necessity for that bane of the profession, *conjecture* or *hypothesis*; and if I were asked whether, if I myself were dangerously ill, I would suffer any hypothetical, however plausible physician, to prescribe for my malady, my answer would be, *No*, assuredly *No*, unless I wished to risk the loss of life. I could give a remarkable instance of this. *Speculation* and hypothesis are always at variance with *sound experience* and successful *practice*."

These quotations are from notes in the Introduction only.

As soon we arrive at page 3 of the work, we have a reference to "History of Physic in Scholæ Medicinæ universalis Nova." In page 6, "My History of Medicine shows much of sects, sectaries, and false systems, &c. with ravings and conceits of every age, &c." In note, page 8, The ingenious Dr. Moseley is introduced with his title of Physician to his *Majesty's Royal* Hospital

 of of Invalids at Chelsea. In page 9, note, the author has the boldness to assert, that there is no danger in natural small-pox if skilfully treated from the beginning, referring us once more to his "Treatises on putrid fevers, &c. Page 11, (note) "There is scarce a week passes that *I* do not prescribe for some miserable case or other." In p. 15, we are referred to the author's "Treatise on malignant and putrid Diseases; and in the text to which this note refers, he expresses his surprise that what he read before the Committee of the Houseof Commons on that subject, is not printed in their minutes. We shall next be taught to wonder that Dr. Squirrell and Mr. Velno's specifics are not ordered to be administered to all who have been vaccinated, or that Dr. Rowley is not consulted by parliament relative to the "modes of treating the beastly new diseases produced from Cow-pox."

If our readers are not tired of this article, we must leave them to labour through the work as we have been obliged to do. It is scarcely necessary to conclude with any remark; but to preserve our customary form, we shall just observe, that throughout the whole the author talks more of himself than of his subject; that those cases which have been proved to be false,* are assumed with as little reserve as if they had been admitted as true; and that all the others, of which we have had leisure to enquire, stand on no better ground. Some instances of failure are doubtless well-founded, and these have been already admitted by the warmest friends of vaccination, Indeed, a true pathologist would be very apt to doubt the truth of any report which boasted infallible success to any practice or discovery. We are pleased, however to find, that, under all these paltry and unworthy attempts to stifle vaccination, it is growing daily more popular; and that all the arguments against it only excite the wished-for enquiry.

---

*The Modern Practice of Physic, by* Edward Goodman Clarke, M. D. *Author of Medicinæ Praxeos Compendium, of the Royal College of Physicians, London.*

We have perused the above work with much gratification, and we earnestly recommend it as deserving of the attention, particularly of the junior branches of the profession, as it is written in an able and scientific manner; and we are well assured that it will not derogate any thing from the reputation the author has already obtained by his Medicinœ Praxeos Compendium, the fourth edition of which is shortly going into the press.

---

* See particularly our Review of Mr. Merriman's Defence, and other articles, as well as original communications in our Journal.

